# Immunological balance between Treg and Th17 lymphocytes as a key element of type 1 diabetes progression in children

**DOI:** 10.3389/fimmu.2022.958430

**Published:** 2022-08-24

**Authors:** Aleksandra Starosz, Milena Jamiołkowska-Sztabkowska, Barbara Głowińska-Olszewska, Marcin Moniuszko, Artur Bossowski, Kamil Grubczak

**Affiliations:** ^1^ Department of Regenerative Medicine and Immune Regulation, Medical University of Bialystok, Bialystok, Poland; ^2^ Department of Pediatrics, Endocrinology, Diabetology with Cardiology Division, Medical University of Bialystok, Bialystok, Poland; ^3^ Department of Allergology and Internal Medicine, Medical University of Bialystok, Bialystok, Poland

**Keywords:** type 1 diabetes, regulatory T cells, Th17 cells, autoimmunity, insulin

## Abstract

Type 1 diabetes (T1D) is autoimmune destruction of the beta cells of pancreatic islets. Due to complexity of that disease, the mechanisms leading to the tolerance breakdown are still not fully understood. Previous hypothesis of imbalance in the Th1 and Th2 cells as the main contributing factor has been recently changed towards role of other lymphocytes – regulatory (Treg) and IL-17A-producing (Th17). Our study aims to assess changes within Treg and Th17 cells in newly diagnosed T1D pediatric patients and their association with disease remission. Flow cytometry implementation allowed for Treg and Th17 analysis in studied groups and further combination with clinical and laboratory data. In addition, expression of diabetes-related genes was tested and evaluated in context of their association with studied lymphocytes. Initial results revealed that Treg and ratio Treg/Th17 are significantly higher in T1D than in healthy controls. Moreover, patients with lower HbA1c and daily insulin requirements demonstrated higher levels of Tregs. Similar tendency for insulin intake was also observed in reference to Th17 cells, together with high levels of these cells in patients demonstrating higher values for c-peptide after 2 years. In low-level Treg patients, that subset correlates with the c-peptide in the admission stage. In addition, higher levels of IL-10 were associated with its correlation with HbA1c and insulin dosage. In the context of gene expression, moderate associations were demonstrated in T1D subjects inter alia between *CTLA4* and Treg or ratio Treg/Th17. Cumulatively, our data indicate a possible novel role of Treg and Th17 in mechanism of type 1 diabetes. Moreover, potential prognostic value of these populations has been shown in reference to diabetes remission.

## Graphical Abstract

The graphical abstract represents the main conclusion of the study. Type 1 diabetic pediatric patients with higher levels of Tregs demonstrated decreased HbA1c and daily insulin requirement values. In addition, patients with increased levels of Th17 cells were shown to have significantly elevated amounts of c-peptide and reduced insulin requirement. Furthermore, remission rates assessment revealed that subjects with increased Treg or Th17 cells demonstrated partial remission more frequently compared to lower levels’ patients. Interestingly, Tregs were found to correlate positively with the CTLA4 expression – associated with excessive immune response control. Thus, possibly reducing autoinflammatory process and leading to higher insulin production, reflected by correlation with c-peptide values in our study. Moreover, Th17 cells were shown to be positively linked to RRAD expression – regulator of insulin tolerance and glucose metabolism. Considering its correlation with daily insulin dose, Th17 were found to affect reduced insulin requirement through RRAD-related mechanisms. Cumulatively, these data shed a new light on Treg and Th17 cells contribution to pathogenesis and progression of type 1 diabetes in children. [Graphical abstract was created with BioRender.com (https://biorender.com) (accessed on 31 May 2022).]

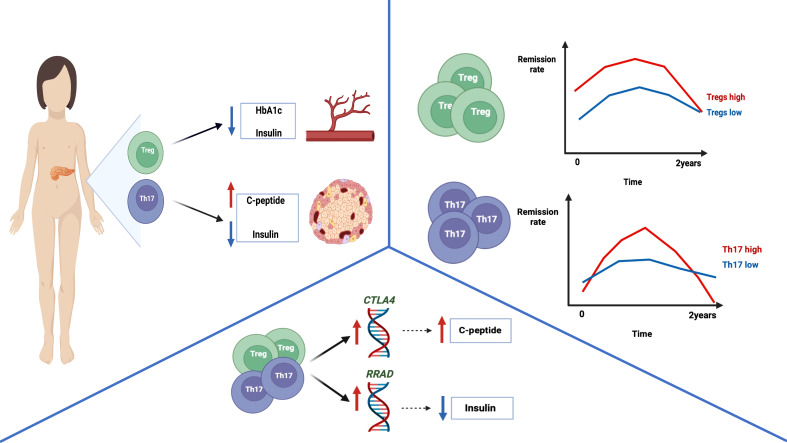

## Introduction

Type 1 diabetes mellitus (T1D), previously known as insulin-dependent diabetes, is a chronic impairment in production or an absolute deficiency of insulin. Its highest prevalence is predominantly associated with children and adolescents. Due to complexity of autoimmune reactions involved in T1D, and additional contribution of genetic and environmental factors, pathomechanism remains a serious challenge. Dominant phenomenon in the course of T1D, responsible for the subsequent symptoms, is an auto-aggressive reaction against pancreatic β cells of Langerhans islets. Subsequent failure of the pancreatic islets leads to a gradual decrease in their number and insulin secretion (1, 2). Autopsy studies indicate that the residual mass of pancreatic islets in patients with T1D is estimate from 2 to 40% (3). Inability to maintain proper glycemia is associated with a numerously significant consequences (4). The disease progression can inter alia contribute to diabetic neuropathy, nephropathy, cardiovascular system disorders, or diabetic retinopathy (5). Therefore, life-long replacement therapy with insulin need to be implemented in those patients (6). Regeneration of pancreatic islets is another key element in the treatment of type 1 diabetes. Recent studies indicate a beneficial effect of very small embryonic-like stem cells (VSELs) on the regeneration of β cells. Increased VSELs cell mobilization correlated with better preserved β cell function by assessing the amount of secreted c-peptide (7). Unfortunately, to date there is no single predictive value that could be used as a reliable parameter for assessing the disease progression. It is worth noting, however, that an enormous scientific progress allowed us to establish genetic risk scores and autoantibody profiles supporting diagnostics and monitoring of the diabetes. State-of-the-art data also indicate the potential role of selected receptors on T lymphocytes that are specific for T1D. These results mention the possibility of developing a biomarker considering cellular parameters and disease activity, particularly on the clonotypes of lymphocytes circulating from the pancreatic tissue into the bloodstream (8). Although numerous factors are considered today as essential in the pathogenesis of T1D, increasing attention is paid to the immunological pathways’ involvement in the endocrine disruption. The occurrence of T1D can be directly linked to breaking the tolerance with infiltration and destruction of the pancreatic insulin-producing cells (6, 9).

Regulatory T cells (Tregs) are a subset of CD4+ T cells exerting suppressive effects on cells. Therefore, they constitute an essential part of self-tolerance mechanisms and the limitation of the inflammatory processes (10). Dysfunction or reduction of these cells might result in a breakdown of immunological tolerance and excessive immune reactions (11). In addition, Tregs function is not solely limited to suppression of T, B or NK cells activity but also participates in tissue repair and regulation of metabolism (12, 13). FOXP3 transcription factor is a crucial component associated with Tregs regulatory properties, and its loss was proved to cause autoimmune disorders – including IPEX syndrome as an example of sole Foxp3 mutation being the main cause (14). A reduced number and impairment of Tregs function have been described in newly diagnosed type 1 diabetes and linked to further disease development (15). Progressive impairment of pancreas may occur without simultaneous clinical manifestation for up to several years, leading to degradation and reduced ability of the pancreatic beta islets to produce and release insulin (16). Decreasing the number of Tregs is an outcome of breaking self-tolerance resulting in the occurrence of pancreatic autoantibodies (17, 18).

Effector IL-17-producing T cells subset, namely Th17 cells, demonstrate an opposite function to regulatory T cells. IL-17A is one of the most significant pro-inflammatory cytokines, best known for its role in other immune cells’ recruitment into a site of an inflammatory process (19, 20). Mutual interactions between Th17 and Treg lymphocytes have been demonstrated previously. While Th17 promotes immune response, Tregs alleviate the symptoms of immune activation (21). The adverse contribution of the Th17 cells and their cytokines, IL-17A and IL-17F predominantly, was found in the context of the T1D progression in human and animal models (22, 23). Studies revealed that expression of Foxp3 inhibited Th17 cell differentiation by blocking the function of specific transcription factors, namely RORγt (retinoid-related orphan receptor gamma t) and RORα (retinoid-related orphan receptor alpha) (24). Nevertheless, RORγt+ Tregs were demonstrated in both, human and animal model. The development of RORγt+-expressing Treg cells depends mainly on the IL6/Stat3 signaling pathway. Mentioned cells, also referred to as Tr17, demonstrate suppressive properties against antigen-specific effector T lymphocytes, thus, suggesting that Tr17 cells might have an ability to reduce autoimmune reactions and inhibit inflammation mediated through Th1/Th17 and Th2 cells (25, 26). Moreover, Th17 cells stimulated by immunological factors might secrete IFN-gamma, which is characteristic for Th1-type cells. Simultaneous production of IL-17 and IFN-gamma cytokines by those cells enhance their pathogenic activity (27, 28). Duality of Th-17-related role in type 1 diabetes pathogenesis has been demonstrated. On one hand, effector Th17 cells reveal a protective effect in an animal model where the immunization process caused an increase in IL-17-secreting cells but did not induce diabetes (29). Additionally, McGeachy et al. proved that Th17 cells differentiated in the presence of TGF-β (transforming growth factor beta) and IL-6 are able to secrete both IL-17 and IL-10. Whereas, sole presence of TGF-β affected the differentiation of primary T cells towards the Tregs (30, 31). Therefore, with demonstrated suppressive potential, these cells may have an impact on preventing diabetogenic processes (32). On contrary, clinical data demonstrated an increasing presence of Th17 and IL-17A and its association with apoptosis intensification and tissue degradation in β-cells of pancreatic islands, thus enhancing the progression of T1D at early stages. Moreover, impairment of Th1/Th17 balance in the peripheral blood together with glucose intolerance was found to reflect the destruction of insulin-producing cells in children with confirmed pancreatic autoantibodies (33).

Despite numerous approaches, the mechanism of immune dysregulation contributing to the T1D onset is still not fully described (34, 35). Disruption of self-tolerance mechanisms is a relevant factor leading to autoimmunity. The immune imbalance between Th17 and Treg cells has been reported previously in autoimmune diseases, including T1D. One of the main factors contributing to that disorder is the increased expansion of Th17 cells with a simultaneous reduction in Treg levels (36). Considering current state of knowledge on Th17 and Treg, it is reasonable to investigate the importance of Treg/Th17 balance in modulation of type 1 diabetes progression. Additionally, verification of these factors in the context of response to treatment and remission would also be an essential element. Accordingly, here we aim to study those aspects in pediatric patients with type 1 diabetes to determine of Treg/Th17 contribution to the disease onset and treatment outcome.

## Materials and methods

### Patients group

The study was conducted on a group of 60 pediatric patients with newly-diagnosed type 1 diabetes mellitus (T1D) treated at the Department of Pediatrics, Endocrinology, Diabetology with Cardiology Division, University Children’s Teaching Hospital Bialystok. Additionally, the control group (HC) was involved constituting 31 pediatric patients with no diabetes and other inflammatory or autoimmune diseases. Patients were enrolled in the study during their hospitalization in the clinical department. For better representation of the untreated group, blood was collected from stable patients, not in the acute state, prior implementation of the subsequent therapeutic regimen. The subjects’ selection criteria were discussed and established together with clinicians. The characteristic of the studied groups was presented in the **Table 1**. Informed consent was obtained from each patient (parent or legal guardian for underage subjects). The Ethical Committee at the Medical University of Bialystok approved the study protocol – APK.002.22.2021.

**Table 1:** The clinical characteristic of the studied groups in context of laboratory parameters - type 1 diabetic pediatric patients (T1D) and healthy control (HC).

Parameter	Type 1 Diabetesmellitus (T1D) Onset	Type 1 Diabetesmellitus (T1D) After 2 years	Control Group (HC)
Age [years]	11.0 (9.0; 13.5)		13.5 (9.5; 14.5)
Body Mass Index [kg/m^2^]	15.80 (14.70; 18.40)	19.30^####^ (17.0; 22.60)	20.30^**^ (16.0; 22.20)
SDS-BMI	-0.500 (-1.11;0.39)	0.463^####^(-0.454;1.273)	0.460^**^ (-0.410;1.150)
Glucose level [mg/dl]	428.5 (296.8; 521.8)		86.0^***^ (80.50; 90.50)
HbA1c [%]	11.46 (10.46; 14.19)	6.86^####^ (6.248; 7.535)	
C-peptide [μ/L]	0.54 (0.34; 0.81)	0.44 (0.155; 0.785)	
GADA [IU/ml]	71.43 (6.55; 289.2)		
IA2A [IU/ml]	437.2 (5.35; 1184)		
ICA [IU/ml]	20 (0; 40)		

Data was presented as a median value with 25th and 75th interquartile range in the brackets. (*- difference between T1D in onset time and HC, # - difference between T1D in onset time and after 2years treatment). The levels of significant differences were indicated with asterisks or exact p values: ** p < 0.01; ***p < 0.001; and octothorpe ####p < 0.0001 respectively.

Patients were included based on the following diagnostic parameters: hyperglycemia (above 200 mg/dL), clinical manifestation and the presence of autoantibodies -GADA (glutamic acid decarboxylase antibodies), IA2A (insulinoma-associated protein 2 autoantibodies), and ICA (islet-cells autoantibodies). Furthermore, body mass index (BMI), normalized body mass index (SDS-BMI), blood glucose levels (Glycemia), glycosylated hemoglobin (HbA1c), and fasting c-peptide were used for evaluation of the patient’s clinical condition. In addition, during 2-years of treatment, the level of glycosylated hemoglobin (HbA1c) and insulin units (daily insulin requirement-insulin dose to achieve normoglycemia in 24 hours), and body weight were measured at the 4 time points (3 months, 6 months, 1 year, and 2 years). The remission stage was assessed by the amount of daily insulin requirement (below 0.5 units/weight) and glycated hemoglobin level (under 7%).

The research material was 4,9 ml of EDTA-anticoagulated peripheral blood collected by venipuncture at diagnosing time. Peripheral blood mononuclear cells (PBMCs) were isolated using density gradient centrifugation with Pancoll 1.077 g/l (PAN Biotech). PBMC cell layers were washed in phosphatate-buffered saline (PBS; Corning) and cryopreserved in fetal bovine serum (FBS; PAN Biotech) with 10% DMSO (Sigma-Aldrich) (stored in liquid nitrogen).

### Flow cytometry

After rapid thawing, cells were resuspended in the culture cell medium RPMI 1640 with the addition of 10% fetal bovine serum. Next, cells were counted on the hemacytometer of the Bürker chamber using the 0.4% trypan blue solution to assess their viability. All the samples used for staining demonstrated viability of 96-100%. The flow cytometric evaluation of Treg and Th17 was performed using 500.000 and 1 million cells, respectively. Noteworthy, further concentration was adjusted and acquired data normalized to include differences in initial cell numbers. Cells for the Th17 panel were stimulated with Leukocyte Activation Cocktail with brefeldin A (BD Pharmingen) for 5 hours at 37°C to increase efficiency of the intracellular cytokines’ detection. Studied patients’ cells were stained with the use of monoclonal antibodies conjugated to fluorochromes aimed at selected surface markers: anti-CD4 FITC (clone RPA-T4), anti-CD25 PE-Cy5 (clone M-A251), anti-CD127 Alexa Fluor 647 (clone HIL-7R-M21), (BD Bioscience). Following incubation unbound antibodies were washed out with PBS. Next, cells were permeabilized using Permeabilization Buffer 2 (BD Bioscience) to allow staining of the intracellular markers with: anti-Foxp3 PE (clone 259D/C7) and anti- IL-17A PE (clone SCPL1362) antibodies (BD Bioscience). Final incubation was followed by wash step and fixation in CellFix reagent (BD Bioscience). Data were acquired with the use of FACS Calibur flow cytometer (BD Bioscience, San Jose, CA, USA). Processing of the flow cytometric data was performed with FlowJo software (Tree Star Inc., Ashland, OR, USA). In context of the gating strategy, lymphocytes were initially distinguished on the basis of their morphology using relative size (forward scatter, FSC) and granularity/complexity (side scatter, SSC) properties. Following selection of the lymphocytes with CD4 marker – Th cells, subsequent markers were analyzed for Treg and Th17 identification. High expression of CD25 and low/negative CD127 marker allowed for regulatory T cells determination. Whereas, Th17 cells were gated on the basis of IL-17A production. Implemented gating strategy, with proper controls applied, was included within **Supplementary Figure 1**.

### Immunoenzymatic assay (ELISA)

Plasma samples collected from patients were used to analyze the presence of the selected cytokines. Immunoenzymatic DuoSet ELISA Kits (R&D system) were implemented in accordance with the manufacturer provided protocol. Tested proteins included those related to the Treg and Th17 cell populations – IL-10 and IL-17 respectively. Cytokine concentration-related absorbance level was acquired at 450nm wavelength using LEDETECT96 microplate reader (Labexim Products, Lengau, Austria). Standard curve based on a four-parameter logistic (4-PL) curve-fit were used to calculate the final concentration of cytokines.

### RNA isolation and RT-PCR

RNeasy Micro Kits (Qiagen) was be used for the isolation of mRNA from the lysate PBMC according to the manufacture protocol. The amount of mRNA was quantified on NanoDrop (Thermo Fisher Scientific; Waltham, MA, USA) by measure the absorbance the width 260 and 280 nm. Assessment was based on the evaluation of the A260/A280 ratio. The correct value was determined in the range 1.8-2.2. Further, the 500 ng of mRNA of each patient was determined to performed the reverse transcription using the High-Capacity cDNA Reverse-Transcription Kit (Applied Biosystems; Foster City, CA, USA) according to the stages: (10 min, 25°C), (120 min, 37°C), (5 min, 85°C). Real-time qPCR was carried using the Human Diabetes Fast TaqMan Assay Kit (Applied Biosystems; Foster City, CA, USA) with the *HPRT1*as a housekeeping gene. The expression of gene: *CD28* (Hs00174796_m1)*, CLTA4* (Hs00175480_m1), *FOXP3* (Hs00203958_m1), *HNF4A* (Hs00230853_m1), *IFNG* (Hs00174143_m1)*, IL10* (Hs00174086_m1), *INSR* (Hs00169631_m1), *NOS3* (Hs00167166_m1), *PPARG* (Hs00234592_m1), *PTPN22* (Hs00247352_m1), *RRAD* (Hs00188163_m1), *HNF1B* (Hs00172123_m1). Stages of reaction are following: 1 cycle of activation of enzyme (95°C, 20s), and 40 cycles of denaturation (95°C, 1s) and annealing with elongation (60°C, 20s), respectively. All assays were performed using the StepOnePlus Real-Time PCR System device (Applied Biosystems; Foster City, CA, USA) and analyzed with manufacture software (StepOnePlus software v2.3). Data was presented as a normalized expression log2 (2^-ddCt) and for correlation was used relative expression (2^-dCt).

### Statistical analysis

Biostatistical analysis of collected data was performed with GraphPad Prism 9.0 statistical software (GraphPad Prism Inc., San Diego, CA, USA) and R studio (RStudio Team (2020). RStudio: Integrated Development for R. RStudio, PBC, Boston, MA, USA). Due to non-Gaussian distribution of the data, non-parametric Mann-Whitney U test was applied to compare differences between groups. Wilcoxon test was used for determination of statistically significant changes in the course of T1D patients’ treatment. Data were presented on the graphs as median values with interquartile range. Significance level was set at p value of 0.05, and differences were indicated with asterisks or exact p values: * - p < 0.05, ** - p < 0.01, *** - p < 0.001, **** - p < 0.0001. Assessment of the correlation between analyzed parameters was performed with nonparametric Spearman correlation test. Data were presented as coefficient values (R value) and significance indicated with asterisks. The following grouping in context of the data strength evaluation was applied: weak (r = 0.20-0.39), moderate (0.40-0.59) and strong (0.60-0.80).

## Results

**Figure 1:**
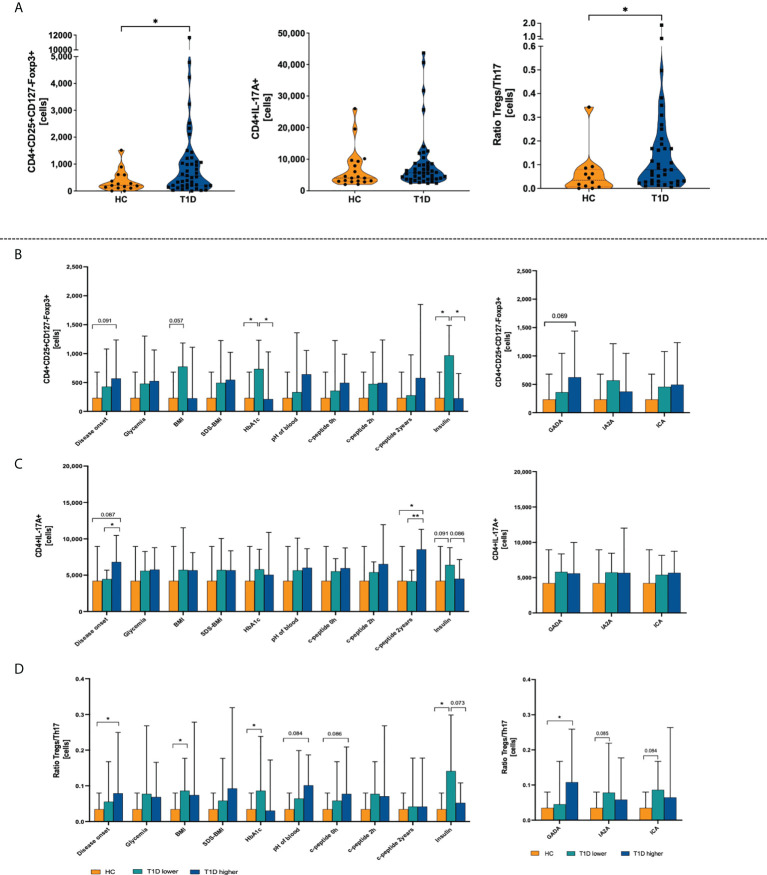
Differences between type 1 diabetes patients (T1D) and healthy control group (HC) in the absolute numbers of regulatory T and Th17 cells. Absolute numbers of T regulatory and Th17 cells, and Treg/Th17 ratio **(A)**. Stratification of patients on the basis on the medium value of clinical parameters (T1D lower-values below the median; T1D higher-values upper median) and demonstration of Treg **(B)**, Th17 **(C)**, and Treg/Th17 ratio **(D)**. The levels of significant differences were indicated with asterisks or exact p values: * - p < 0.05; ** - p < 0.01.

Initial analysis revealed that T1D patients are characterized by significantly higher absolute counts of regulatory T cells than the healthy control group subjects. No changes were found in the context of IL-17A producing Th17 cells-producing Th17 cells. In accordance, disturbance in Treg versus Th17 immune balance, here defined as Treg/Th17 ratio, seems to be associated with changes in the Treg population in pediatric patients (**Figure 1A**). Subsequently, we analyzed the immune data using T1D patients’ stratification on the basis of median of clinical/laboratory procedures results at the diagnosis time (“T1D lower” included patients whose value of the clinical parameter was below the median and “T1D higher” represents patients with values upper the median). Interestingly, group with higher age of the disease onset demonstrated significantly higher levels of Th17 compared to those with earlier diagnosis. We did not find differences in Tregs or Th17 cells in context of glycemia at admission. However, diabetic patients with lower HbA1c values were shown to have significantly elevated numbers of Tregs compared to those with worse long-term control of the glycemia. The same observations have been made in reference to insulin dose (daily insulin requirement, DIR) where those with lower insulin dosage (DIR) demonstrated increased levels of regulatory T cells. Essential differences regarding Tregs were also found in autoantibodies-based stratification. (**Figures 1B, C**) All these changes were reflected by Treg/Th17 balance-related ratio. In addition to above data, higher values of the ratio were observed in patients with high levels of GADA autoantibodies (**Figure 1D**).

**Figure 2:**
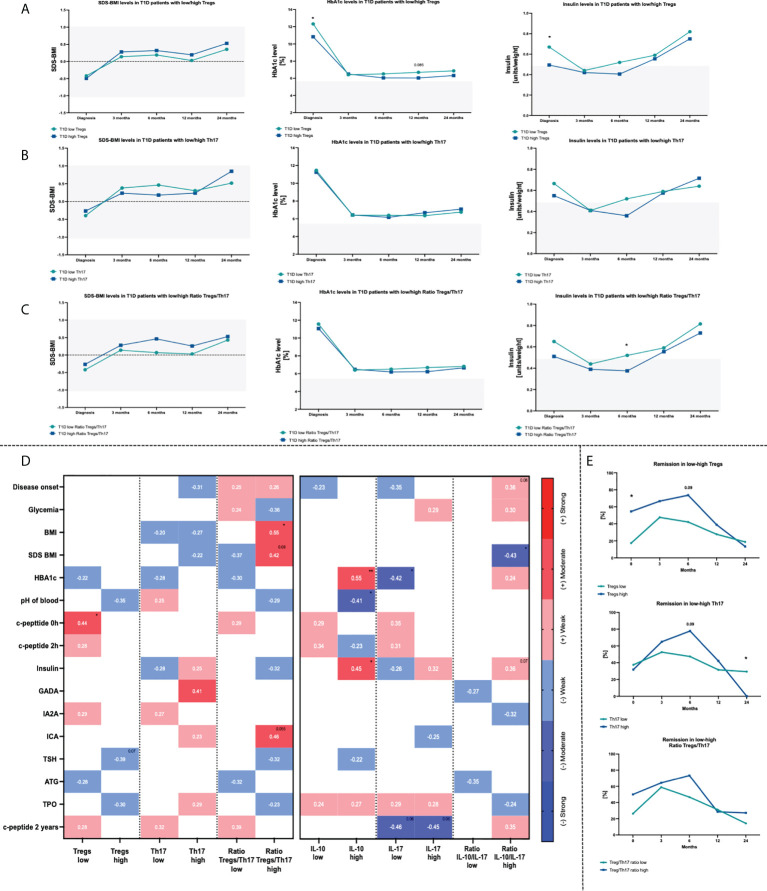
Influence of Treg and Th17 cells level on changes within HbA1c, SDS-BMI and Insulin dosage. Time-related variations in selected parameters in context of Treg **(A)**, Th17 **(B)** cells and Treg/Th17 ratio **(C)**. Correlations between Treg/Th17 cells or related plasma cytokines (IL-10/IL-17A) and diagnostic data **(D)**. Frequency of T1D children in remission state depending on the initial Treg or Th17 cells numbers **(E)**. Heat-maps demonstrate r values with exact values and color indicating strength subgroup. The levels of significant differences were indicated with asterisks or exact p values: * - p < 0.05; ** - p < 0.01.

Standardized BMI (SDS-BMI), glycated hemoglobin (HbA1c) and insulin dosage per weight were monitored in the studied patients. T1D subjects were divided into two subgroups based on the median value of the Tregs, Th17 cells or Treg/Th17 ratio in each group (“T1D low Treg/Th17 or Ratio Treg/Th17” when the absolute cell number was below the median value and “ T1D high Treg/Th17 or Ratio Treg/Th17” when the absolute cell number was upper than median value), to verify their significance in the course of therapy. First, we found that patients with lower Treg levels demonstrated initially higher values for HbA1c and insulin dose. These differences were reduced later as both groups responded similarly to the treatment applied (**Figure 2A**). No significant changes were shown in reference to Th17 cell levels and selected parameters. However, slightly higher initial values of HbA1c and 6^th^ month insulin doses were found in T1D patients with lower Th17 cells (**Figure 2B**). Variations in both, Treg and Th17 cells led to higher insulin doses in low Treg/Th17 ratio patients after 6 months of therapy (**Figure 2C**).When analyzing the association between studied immune cells and diagnostic parameters, we found a moderate correlation between Tregs and c-peptide in patients with low level of these cells (data statistically significant, p < 0.05). Similar strength of association was found between Th17 cells in high-level group and GADA autoantibodies. In addition, we showed that a moderate negative correlation could be attributed to Tregs in subjects with high levels and TSH activity (p = 0.07) (**Figure 2D**). Treg and Th17 cells were found to contribute essentially to the disease remission in the T1D patients. Subjects with higher levels of Tregs demonstrated more patients at remission state compared to the opposite group. Despite the fact that these differences diminished at the 24^th^ month of therapy, frequency of patients with remission remained higher approximately up to 12 months. Regarding Th17 cells, T1D individuals with higher levels of that cell population showed a higher remission rate between 3^rd^ and 12^th^ month of treatment. Treg/Th17 ratio demonstrated direction of changes similar to Tregs in the context of remission frequencies, however, at less pronounced rate (**Figure 2E**).

**Figure 3:**
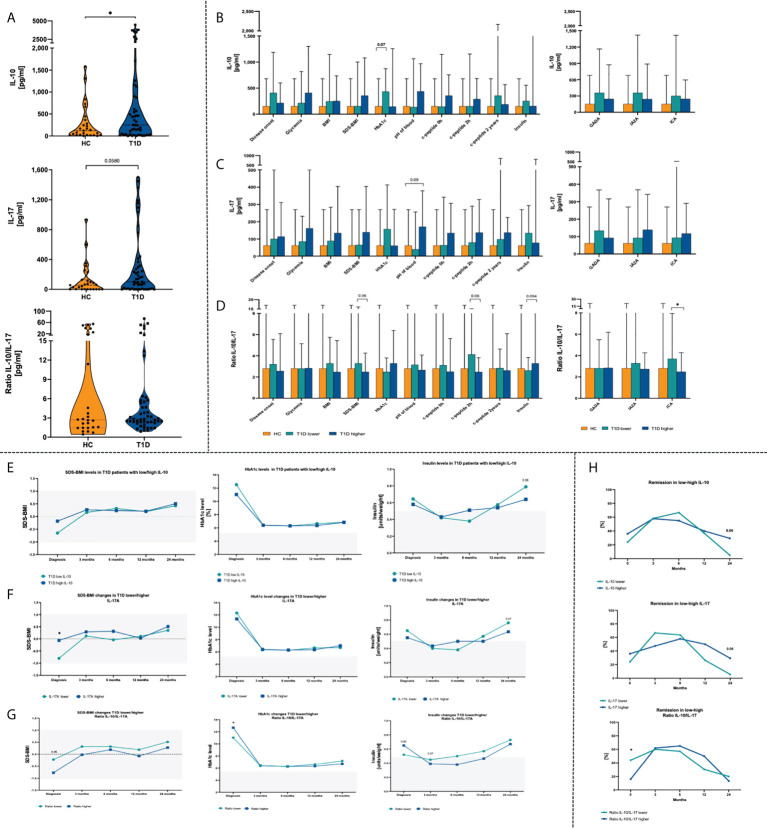
Changes in plasma cytokines in the T1D patients. Admission stage values of IL-10, IL-17 and IL-10/IL-17 ratio **(A)**. Effects of IL-10 **(B)**, IL-17 **(C)** and their ratio **(D)** on the clinical parameters in the studied groups. Time-related variations in SDS-BMI, HbA1c and insulin dosage in context of low/high IL-10 **(E)**, IL-17 **(F)** and IL-10/IL-17 ratio **(G)**. Variations in remission rates depending on IL-10 and IL-17 T1D patients **(H)**. The levels of significant differences were indicated with asterisks or exact p values: * - p < 0.05.

Subsequent analysis of plasma cytokines revealed that interleukin 10 (IL-10) and interleukin 17 (IL-17) are in accordance with Treg and Th17 cells. Higher level or tendency was demonstrated for IL-10 and IL-17 respectively in T1D patients compared to healthy group. We did not observe differences in case of IL-10/IL-17 ratio (**Figure 3A**). Stratification of T1D patients into those with low/high levels of tested cytokines or their ratio allowed to evaluate their influence on laboratory results. Patients with lower concentration of IL-10, similarly to Treg, showed higher levels of HbA1c. Similar tendencies were found in context of SDS-BMI, c-peptide and ICA autoantibodies in subjects with low Treg/Th17 ratio (**Figures 3B–D**). Assessment of the laboratory parameters in time that patients with a lower baseline plasma level of IL-10 had a tendency (p = 0.08) for higher insulin doses required at 24^th^ month of therapy (**Figure 3E**). Likewise, a similar trend was observed with the IL-17 levels (p = 0.07). In addition, those with high plasma IL-17 concentration had significantly initially higher SDS-BMI values (**Figure 3F**). T1D patients with higher IL-10/IL-17 ratio demonstrated elevated HbA1c and insulin dosage at the admission stage. These differences, however, diminished in the course of treatment implementation (**Figure 3G**).

Subsequently, correlation of plasma cytokines with laboratory parameters was performed. High-level IL-10 patients demonstrated moderate strength positive association of that cytokine with HbA1c (p < 0.01) and insulin requirement (p < 0.05), and negative in reference to blood pH value (p < 0.05). In contrast, T1D subjects with lower concentrations of IL-17 initial levels of IL-17 correlated negatively with HbA1c (p < 0.05) and value of c-peptide at 24^th^ month of the therapy (p < 0.05). Interestingly, association between IL-17 and 24^th^-month c-peptide was shown in both, low- and high-level groups. IL-10/IL-17 ratio was noticed to correlate negatively with the SDS-BMI values (p<0.05). Regarding insulin dose and age of the disease onset, a weak positive correlation was found in patients with higher IL-10/IL-17 ratio (**Figure 2D**).Remission rates analysis in T1D patients revealed that group with lower IL-10/IL-17 ratio had comparably higher percentage of subject with remission at initial stage. Furthermore, those with initially lower levels of plasma IL-17 had achieved higher remission rates already at 3^rd^ month of therapy. However, in the subsequent months these values dropped considerably. Both, low IL-10 and IL-17 patients at 24^th^ month of treatment implementation demonstrated lower percentage of subjects with remission that those with higher levels of these cytokines at the admission stage (**Figure 3H**).

**Figure 4:**
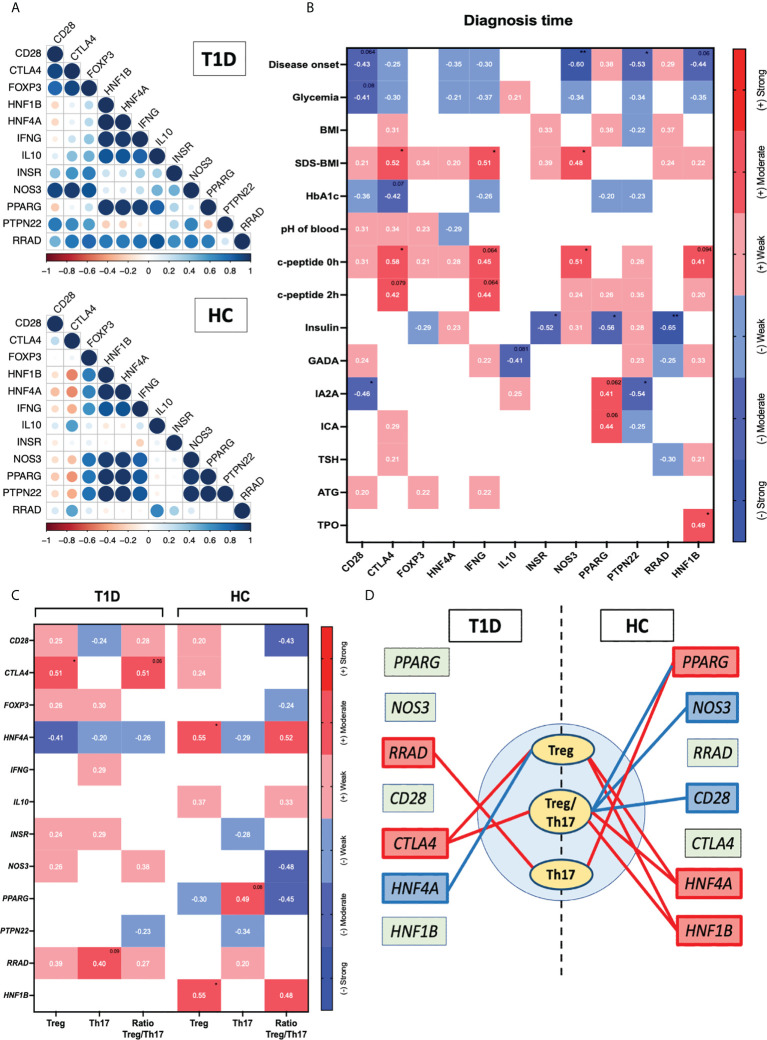
Assessment of diabetes-related genes association with the immune cells. The mutual interactions of selected genes in healthy control and T1D group (r values represented by circles diameter) **(A)**. Correlation of the genes with the laboratory parameters at time of diagnosis **(B)**. Demonstration of Tregs, Th17 and their ratio relation to the tested genes **(C)**. Graphical visualization of most essential connections between the immune cell subsets and diabetes-related genes (positive/negative correlations indicated with red/blue color respectively; strength based on r values demonstrated through the lines width) **(D)**. Heat-maps demonstrate r values and color indicating strength subgroup. The levels of significant differences were indicated with asterisks or exact p values: * - p < 0.05; ** - p < 0.01.

Next, we intended to evaluate association between expression of genes related to diabetes and selected immune cell populations. First, we noticed that the mutual correlations pattern differed between T1D patients and healthy controls in clustering analysis. Those differences could be seen especially in reference to *PTPN22*, *IL10*, *INSR*, *IFNG* and *RRAD* (**Figure 4A**). Furthermore, the mutual association the *CD28* as well as *CTLA4* and *FOXP3* does not occur in healthy patients but is observed in T1D patients. Furthermore, we demonstrate the moderate negative association between *CTLA4* and other analyzed genes, and the mentioned correlation is absent in then T1D group. Additionally, several of that genes were found to correlate with laboratory parameters at time of diagnosis. *RRAD* was demonstrated inter alia to have a strong negative association with insulin dose (p < 0.01). Negative correlation with insulin applied was also reported in reference to *PPARG* gene (p < 0.05). That expression was also associated positively with the level of IA2A and ICA autoantibodies (p =0.062 and p=0.06). Another gene – *PTPN22*, was shown to correlate negatively with the disease onset (p < 0.05), and IA2A (p < 0.05). Similar interaction with onset of the disease was found in context of *HNF1B* gene (p < 0.062), c-peptide (p=0.09) and TPO value (p<0.05). Interestingly, we found a moderate positive correlation fasting c-peptide and after 2 hours with the expression of *CTLA4* (p<0.05 and p=0.079 respectively) and *INFG* (p=0.064 and p=0.064). These genes were also indicated in association with the SDS-BMI value (p<0.05). Moreover, the expression of *CTLA4* presents a correlation with the glycated hemoglobin level (p=0.07). The *CD28* gene expression was negatively associated with the age of disease onset (p=0.064), level of glycemia (p=0.08) and IA2A antibody level (p<0.05). Finally, *NOS3* was found in a strong negative association with the age of disease onset (p<0.01) and a moderate positive correlation with the SDS-BMI and fasting c-peptide (p<0.05). (**Figure 4B**).

Correlation analysis of selected genes and the immune cell populations revealed significant changes between T1D and healthy control groups. Regarding Tregs, in the HC group positive correlation with all the following genes was found: *HNF4A* and *HNF1B* (p < 0.05). On contrary, diabetic patients demonstrated correlations with *CTLA4* (p < 0.05) and also with *HNF4A* however, those association were negatively compared to HC. Positive moderate correlation with *RRAD* was also showed in reference to Th17 cells (p < 0.09). Interestingly, in healthy controls Th17 subsets were associated positively with *PPARG* what was diminished in T1D patients. In addition, negative correlation Ratio Treg/Th17 in control group cells with *CD28*, *NOS3* and *PPARG* and positive with *HNF4A* was also not demonstrated in diabetic subjects (**Figures 4C, D**). The most relevant correlations between immune cell subsets and diabetes-related genes were presented as a graphic visualization (positive/negative correlations indicated with red/blue color respectively; strength based on r values demonstrated through the lines width). (**Figure 4D**)

## Discussion

Type 1 diabetes as an autoimmune disease is characterized by an impairment of the immune balance. Thus, resulting breakdown of the immune tolerance mechanisms leads to the gradual destruction of the pancreatic beta cells. Regulatory T cells are responsible for maintaining self-balance and preventing immune system hyperactivity through suppressive activity. Role of the CD4+Foxp3+ cell in preventing type 1 diabetes was demonstrated by Feuerer et al. using the NOD (non-obese diabetic) mice model (37). Unfortunately, the proper function of Tregs is diminished completely or partially effective in T1D. On contrary, Th17 lymphocytes over-reactivity was found to be associated with inflammation promotion and even insulin resistance (22). Due to complexity of immune-related pathomechanism in insulin-dependent diabetes, researchers worldwide are still trying to provide complete explanation of the condition.

Type 1 diabetes is a disease accompanied by numerous immunological processes which through changes in cellular compartments, lead to strengthening or suppression of the immune responses. In the first observations, our research group demonstrated elevated levels of T regulatory cells compared to the healthy control group. Previous data indicated a decrease in the frequency of Treg together with an increase in Th17 cells, especially at early stage of the disease (36, 38). Accordingly, here Th17 demonstrated slightly higher levels, supported additionally by tendency for elevated IL-17 concentration in plasma of T1D patients. We presume that such contrasting data, especially in reference to Treg levels, might be associated with individual variabilities and level of heterogeneity of T1D (39). Nevertheless, despite the average level of Treg cells in diabetic patients, these cells may have abnormal functionality, inter alia, in response to IL-2 stimulation. Moreover, effector lymphocytes of people with developing diabetes are more resistant to Treg suppression (40). Attempts to assess the functionality of Treg in diabetic patients indicate both impairments of their functioning and resistance to suppression in effector T lymphocytes (41). Additionally, impaired Treg function and an increased response to suppression have been observed in the so-called slow progressors. Nevertheless, the abnormal Treg function can be overcome by increasing the ratio of Treg to effector lymphocytes (42). Moreover, lack of significant changes in the circulating peripherally Th17 cells might also be associated with enhanced infiltration of Th17 into inflammatory tissue of the pancreas. Residual IL-17-secreting cells attract additional pro-inflammatory cells, such as neutrophils and plasmacytoid dendritic cells, into site of pancreatic inflammation (43). Moreover, we must remember that our study implemented a more precise regulatory T cells phenotype - CD4+CD25+CD127-Foxp3+, and analysis of absolute cell numbers instead of frequencies. In accordance with our data, higher Tregs have already been demonstrated in type 1 diabetic subjects in different study groups (15, 35). Noteworthy, some studies showed cells with regulatory phenotype to exhibit disturbances or even complete lack of their function (44). Considering concomitant higher levels of IL-10 in plasma of T1D patients, an increase in regulatory T cells could be a compensatory mechanism counteracting excessive inflammatory process accompanying the disease. In fact, resident pancreatic Tregs play a significant role in suppressing inflammatory activity and proliferative infiltration of autoreactive CD4+ and CD8+ lymphocytes. Moreover, Tregs demonstrated cellular and humoral - IL-10- and TGF-β-based mechanisms in their anti-inflammatory activity (45, 46). Therefore, we observed Tregs and IL-10 elevation in studied patients. Moreover, IL-10 was previously reported as associated with preventing diabetes by restraining the insulitis and improving beta cell condition (47). In addition, we hypothesize that no essential changes in Th17 cell and their dominant cytokine might be an early sign of the suppressive function efficiency. But, Tregs may contribute to an increase in IL-17A, since cytokine-producing Tregs like Th1 and Th17 have already been described. This may indicate that these pro-inflammatory cytokines produced by them may be essential for immunosuppressive activity (48).

Demonstrated variations in Treg and Th17 cells, and their corresponding cytokines, required further investigation of their contribution to the diabetes-related parameters. That was possible through stratification of T1D patients into those with low or high levels of selected laboratory data. First, we showed that groups characterized by lower HbA1c or insulin dose in ITT additionally demonstrated higher absolute numbers of regulatory T cells. Patients with an initially higher number of Tregs maintained lower insulin dose requirements up to 6^th^ month of therapy. We observed a similar relationship in case of Th17 and Treg/Th17 cells ratio. Furthermore, decline in remission frequency among patients from the 6^th^ month of therapy was observed previously by Klocperk and it was related to an increase in daily insulin administration (49). In our study group we found that key point of changes in insulin intake seemed to be related to Th17 and Treg/Th17 cells ratio levels. In addition, we also showed that after 6^th^ month remission rates starts to drop down, and were observed to be associated with Treg and Th17 cells numbers. Reduced levels of IL-17-producing lymphocytes were associated with earlier onset of the insulin-dependent diabetes, and c-peptide decline at 24^th^ month of therapy. Those results are in consent with previous experimental study where administration of autologous Tregs was associated with further increase in c-peptide and reduction of insulin demand (50). In addition, in subjects with high GADA autoantibodies, increased Treg/Th17 ratio was found – resulting from elevated Tregs and slightly reduced Th17 cells. Thus, indicating crucial role of improper balance of Tregs to Th17 in diabetes-related autoimmune process rather that individual changes in each cell subset.

Initial associations between laboratory results and Treg or Th17 lymphocytes provoked a question on these subsets admission levels influence on the course of the diabetes therapy. At diagnosis, higher HbA1c levels and daily insulin doses were reported in patients with low levels of regulatory T lymphocytes. However, implementation of treatment led to essential reduction of these differences where at 24^th^ month low/high Treg or Th17 groups demonstrated the same levels of the selected parameters. Additionally, plasma IL-10 and IL-17 results were also in accordance with immune cells data, with only tendencies in groups with low levels of these cytokines for higher doses of insulin required at the end of therapy. The important role of suppressive cells, and secreted cytokines such as IL-10, has been well described to date. In relation to our study, their critical participation was observed inter alia in NOD model where increased levels were associated with delaying autoimmune disease development (51). Considering here observed an increase in Tregs, its possibly affected properties might have led to such changes to allow immune balance control despite disrupted cell function. That hypothesis is supported by demonstrated fact that expanded Tregs also initiated higher production of IL-10. IL-10 overexpression may rapidly induce even the amount of Treg cells, thus contributing to a milder course of autoimmune diseases, including type 1 diabetes (52). Nevertheless, increased expression of IL-10 on pancreatic cells was also recognized as a negative predictor of disease intensification resulting from the simultaneous activation of ICAM-1 on endothelial cells, and subsequently, disease progression through recruitment of inflammatory cells (53, 54).

Apart from monitoring of the disease-related parameters in the course of treatment application, we also verified direct associations between tested immune cells (and their cytokines) and laboratory results. In low-level Treg patients, cells with regulatory properties were found to correlate significantly with c-peptide at admission stage. Previously, Tregs reduction was found to be associated with worse disease control due to a simultaneous increase in the level of glycosylated hemoglobin (55). In our study, we observed a similar tendency as patients with initially higher Tregs levels demonstrated better control of glycemia reflected by HbA1c reduction and enhanced sensitivity to exogenous insulin. In accordance, despite type 1 diabetes-related breakage of tolerance T cells with suppressive potential still maintain function required for the disease control. The observed positive correlation between low Treg values and high c-peptide levels at admission time strengthens our hypothesis of incomplete loss of suppressive abilities. Interestingly, in those with higher IL-10 concentrations, that cytokine showed positive association with HbA1c and daily insulin dose requirement. Thus, these data might support previous reports on ambiguous nature of IL-10 – on one hand, preventing excessive immune reactions, but on the other, leading to promotion of inflammation and progression of the disease (53, 54). On the contrary, IL-17 plasma concentration was found to negatively correlate not only with initial HbA1c level, but also with c-peptide at 24^th^ month of therapy. However, as these relations are diminished when IL-10/IL-17 ratios are analyzed, we can presume that proper immune balance is most crucial in the process of the insulin-dependent diabetes control.

An interesting phenomenon documented in the literature, is a partial transient remission characterized by a reduced demand for exogenous insulin and a significant reduction in glycated hemoglobin level (56). In our study, we were observing that clinical situation between 3^rd^ and 6^th^ month of therapy. Subsequently, however, number of patients in remission have gradually decreased in time. Unfortunately, the mechanisms controlling partial remission of the disease are still unknown. Various literature examples of attempts can be described that tried to establish association between partial remission and numerous cellular and biochemical parameters. It has been shown inter alia that patients with a higher frequency of CD4+CD25+CD127high lymphocytes at the onset of the diabetes, exhibited more extended period of remission during treatment. In addition, those patients’ regulatory cell levels remained unchanged compared to the non-responder group (57, 58). Furthermore, Gomez-Munos et al. demonstrated increased levels of IL-10 and IL-17 in type I diabetic patients at the time of diagnosis, however, they did not find a significant impact of these on the incidence of the partial remission (56). Initial levels of the immune cells and corresponding cytokines were not neutral to the remission rates of our diabetic patients. Those subjects with higher Tregs and Th17 cells predominantly were showed to have higher remission rates up to 12^th^ month of the therapeutic approach. After that time, the differences started to diminish and both, low- and high-level Treg or Th17 patients exhibited comparable remission rates. IL-10 or IL-17 did not demonstrate the same pattern, however, high values of these cytokines in T1D patients were followed by tendency for better remission rates even at the end of the disease monitoring. Nevertheless, the percentage of patients with initially lower levels of IL-17 achieved partial remission in a greater number of cases at the 3^rd^ month of therapy.

Assessment of genes linked to the type 1 diabetes pathogenesis revealed different pattern of mutual correlation and clustering between selected genes, within studied patients and control group. In further analyses we found that selected immune cell populations are linked to the tested genes to a various extent. Among all reported results, most significant observations seemed to be associated with *RRAD* gene that was found to strongly correlate with Th17 cells in T1D patients. Noteworthy, *RRAD* coding Ras-related GTP-binding proteins was shown in the past to contribute to glucose uptake reduction in response to insulin, thus, leading to insulin resistance (59). Moreover, its activity was also found to affect proliferation and differentiation of cells (60). Upregulated expression of *RRAD* was found in the islet tissue of healthy people stimulated by type 1 diabetes patients serum (61), and in the muscle tissue of type 2 diabetes patients (62). Interestingly, it was observed that *RRAD* expression could be positively regulated by insulin presence. In addition, insulin resistance was demonstrated to coexist together with overexpression of *RRAD* in isolated muscle cells (63). Increased expression of *RRAD* has already been widely documented in type 2 diabetes, however, our results indicate that it may also have a significant impact on the pathogenesis of insulin-dependent type of diabetes. First, negative strong correlation was found between the relative expression of *RRAD* and the level of insulin, indicating an existence of its association with reduction of insulin production by damaged pancreatic islets. In addition, strong positive correlation of *RRAD* with number of Th17 cells can partially explain the initial period of the disease. At that time, increased infiltration of Th17 cells as a result of damage to pancreatic islet cells by autoantibodies and the constantly increasing levels of Treg cells responsible for suppressing excessive immune reactions were observed. Interestingly, Th17 cells of healthy control group demonstrated moderate association with *PPARG* gene. Interestingly, however, ratio Treg/Th17 represents a negative correlation with that gene. Increased expression of *PPARG* (peroxisome-proliferator-activated receptor gamma) was attributed to intensified adipogenesis, but most importantly to elevated sensitivity to the insulin (64). In addition, Maganti et al. proved that activation of *PPRAG* significantly influences the function of pancreatic beta cells in the NOD model, and even the survival of these mice. The mentioned process also resulted in an increased levels of insulin in the blood serum, but did not induce T cells within the pancreas (65). Importantly, in our study group, we observed strong positive correlations between the relative expression of *PPRAG* and pancreatic autoantibodies. Despite the effects of *PPARG* observed by Maganti, here using the mRNA of peripheral blood cells, we observed that its increased expression significantly influences the levels of released autoantibodies. Current scientific reports indicate two potential models taking into account the role of *PPARG* in disease progression. The first one assumes a beneficial effect of *PPARG* activation on the increase of cells’ sensitivity to insulin. The second one assumes the direct variability of the mentioned gene on pancreatic islet cells. Increased activation of *PPARG* in the initial stage may contribute to faster regeneration of beta cells and thus inhibit the disease progression, or even allow to achieve a remission state (66). Regarding Th17 lymphocytes, we observed its essential association with *PPARG* and only weak correlation with *INSR* present in healthy control group, and highly relevant correlation with *RRAD* only in T1D subjects. Noteworthy, receptor for insulin (coded by *INSR*) is not only responsible for the glucose metabolism-related phenomenon but also participates in promotion of T cell proliferation and effector cells support. In accordance, *INSR* may additionally control T cell activation *via* modulation of their metabolism (67). The CTLA-4 is an inhibitory receptor on the surface of lymphocytes, which is necessary for the maintenance of self-tolerance and homeostasis within T lymphocytes. CTLA-4 demonstrates dual role in immunity, inhibiting inappropriate activation of naive T lymphocytes (Treg) and preventing the accumulation of autoreactive T lymphocytes in tissues (68). Numerous single nucleotide polymorphisms have been described as affecting the level of CTLA-4 on Treg cells and thus affecting their functionality (69). Administration of CTLA-4-Ig can delay β- cell impairment and disease progression. The obtained response was based on blocking CD28 and CD3 costimulatory molecules and impairment of autoactivation of reactive T cells (70). In reference to recent data, we demonstrated the positive strong correlation between *CTLA4* mRNA expression with fasting and stimulated c-peptide suggesting its crucial contribution. Interestingly, we found its direct association with Tregs, therefore, we believe that *CTLA4* within these cells might be one of the key elements in autoimmune phenomenon accompanying T1D. In contrary, another member of checkpoint proteins family - *CD28*, showed negative correlation with IA2A Abs levels and glycemia. These observations are also supported by previously reported relation of CD28 expression on T cells with the disease duration (71). Therefore, the data might indicate that the mechanisms of restoring the immune balance are still functioning partially despite significant disturbances in glucose metabolism (72).

## Conclusion

Type 1 diabetes mellitus is an impairment of insulin secretion caused by autoimmune destruction of Langerhans β islets of the pancreas. Recent hypotheses on the outcome of T1D focused on the imbalance of mutual interaction between suppressive regulatory T cells and effector Th17 lymphocytes. Here we managed to assess participation of these two populations in newly diagnosed pediatric patients with T1D. We demonstrated involvement of Treg and Th17 cells in disease-related parameters, namely: HbA1c, insulin requirement and c-peptide. Most importantly, levels of the studied immune cells were closely related to the remission rates in the course of drug intervention. We indicated that changes in Tregs might be associated with balancing autoimmune process, supported additionally by enhanced immunosuppressive function of these cells. Furthermore, we demonstrated for the first time that Th17 cells correlate significantly with *RRAD* in the course of T1D and *PPARG* in HC group. Expression of these genes was previously linked to the regulation of insulin tolerance and glucose metabolism. In fact, our study revealed that higher levels of Treg and Th17 cells might be associated with higher expression of *RRAD*, thus reducing daily insulin dose. Importantly, analysis of *CTLA4* and *CD28* genes revealed association of these checkpoint proteins not only with the disease-related parameters, but also in reference to Tregs. In conclusion, regulatory and IL-17-producing cells seems to play crucial role in the course of type 1 diabetes. Subsequent studies aimed at Tregs-related molecules found there – namely CTLA-4, CD28 and coded by *RRAD*, might allow for in-depth verification of possible targets for better immune tolerance control. Novel pathways of interactions demonstrated here shed a new light on the Treg/Th17 balance effect on the disease pathogenesis and progression. Most importantly, we provided direct insight into Tregs and Th17 cells association with the outcome of T1D therapy, thus, contributing significantly to an increase in a clinically practical knowledge. Further research would allow to find direct application for these results in context of T1D monitoring or even therapeutic approaches.

## Data availability statement

The raw data supporting the conclusions of this article will be made available by the authors, without undue reservation.

## Ethics statement

The studies involving human participants were reviewed and approved by Ethical Committee at the Medical University of Bialystok approved the study protocol – APK.002.22.2021. Written informed consent to participate in this study was provided by the participants’ legal guardian/next of kin.

## Author contributions

KG and AS have made substantial contribution to the project concept. KG and AS have designed the work and supervised project implementation. KG, AS, BG-O and MJ-S have performed the experiments, acquired and analyzed data. KG, AS, MM and AB summarized the data and prepared the manuscript. All authors read and approved the final manuscript. All authors contributed to the article and approved the submitted version.

## Funding

This research was funded with subvention of Medical University in Bialystok No SUB/1/DN/21/004/1199.

## Conflict of interest

The authors declare that the research was conducted in the absence of any commercial or financial relationships that could be construed as a potential conflict of interest.

## Publisher’s note

All claims expressed in this article are solely those of the authors and do not necessarily represent those of their affiliated organizations, or those of the publisher, the editors and the reviewers. Any product that may be evaluated in this article, or claim that may be made by its manufacturer, is not guaranteed or endorsed by the publisher.

## Supplementary material

Supplementary Figure 1 Gating strategy used in flow cytometric analysis of Tregs and Th17 cells. Analysis of Th17 cells was based on detection of IL-17A production within CD4+ lymphocytes (A). Regulatory T cells (Treg) were distinguished using presence of CD25+CD127- phenotype and presence of Foxp3+ expression within CD4+ lymphocytes (B). All the necessary FMO and ISO controls implemented are demonstrated.
