# Supplementary figures and images for: Immunological balance between Treg and Th17 lymphocytes as a key element of type 1 diabetes progression in children
Source: Front Immunol. 2022 Aug 24;13:958430. doi: 10.3389/fimmu.2022.958430 (PMC9449530; doi:10.3389/fimmu.2022.958430)

**A**

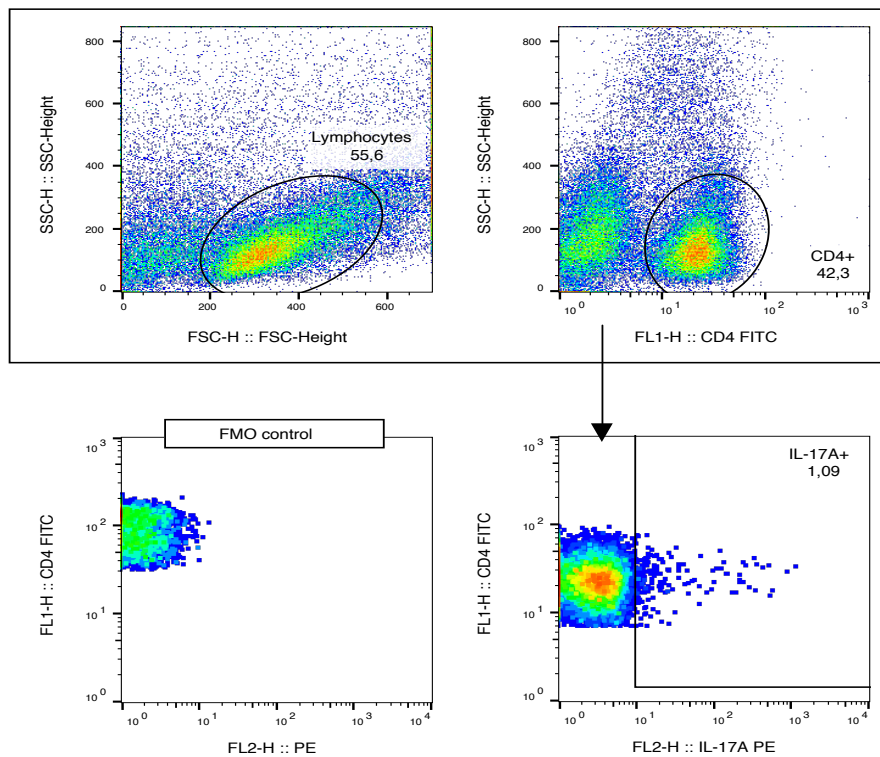

**B**

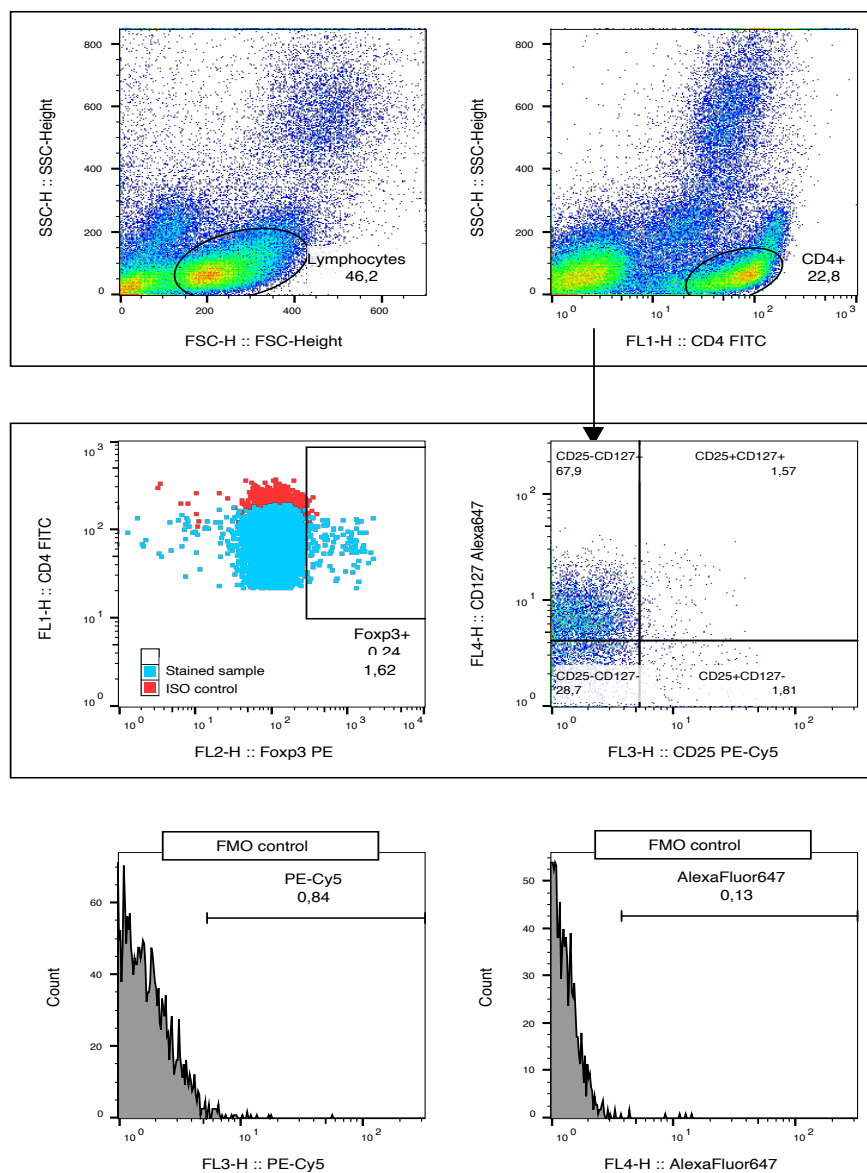

Supplement: Supplementary Figure 1 — Gating strategy used in flow cytometric analysis of Tregs and Th17 cells. Analysis of Th17 cells was based on detection of IL-17A production within CD4+ lymphocytes (A). Regulatory T cells (Treg) were distinguished using presence of CD25+CD127- phenotype and presence of Foxp3+ expression within CD4+ lymphocytes (B). All the necessary FMO and ISO controls implemented are demonstrated. [file DataSheet_1.pdf]
